# Epidemiology of laboratory confirmed measles virus cases in the southern nations of Ethiopia, 2007–2014

**DOI:** 10.1186/s12879-017-2183-5

**Published:** 2017-01-19

**Authors:** Mekonen Getahun, Berhane Beyene, Ayesheshem Ademe, Birke Teshome, Mesfin Tefera, Aklog Afework, Yoseph HaileMariam, Esete Assefa, Yonas Hailegiorgis, Anjelo Asha

**Affiliations:** 1 Ethiopian Public Health Institute, Arbegnoch Street, P. O. Box 1242, Addis Ababa, Ethiopia; 2 WHO Country Office, Addis Ababa, Ethiopia

**Keywords:** Measles, SNNPR, Ethiopia, 2007–2014

## Abstract

**Background:**

In Ethiopia, measles case-based surveillance was introduced in 2004 as one strategy for measles control by laboratory confirmation of suspected cases. In this article, epidemiological distribution of laboratory-confirmed measles cases were reported from the Southern Nation Nationalities and Peoples Region (SNNPR) of Ethiopia between 2007 and 2014, as the region is one of the highly measles affected areas in Ethiopia.

**Method:**

A serum sample was collected from all measles suspected cases, and patient information was captured by case reporting format (CRF). Samples were transported to the National Measles Laboratory for Measles IgM testing by ELISA technique. Data entry and analysis were done using Epi-Info 3.5.4 software.

**Result:**

A total of 4810 samples were tested for measles IgM using ELISA technique and 1507 (31.3%) were found positive during 2007–2014 in SNNPR of Ethiopia. Patients with age 1–4 years were the most affected regardless of sex. The incidence of measles confirmed cases increased from 15 in 2007 to 180 in 2013 per million population. The highest percentage of laboratory-confirmed cases were found in 2014. Measles was found distributed throughout the regional state.

**Conclusion:**

Measles was found a public health important disease in SNNPR of Ethiopia, mostly affecting children 1–4 years. The incidence of measles cases is increasing from time to time. Additional research to determine the genotype of circulating measles virus, knowledge, attitude and practice of professionals and the population for measles vaccination and infection in the region is important. A wide age group measles vaccination campaign is highly recommended.

## Background

Measles is an acute, highly infectious viral disease caused by *morbillivirus* in the family Paramyxoviridae for which humans are the only reservoirs. The primary transmission is person-to-person via aerosolized droplets or by direct contact with nasal and throat secretions of infected persons. When measles virus infect a non-immune population, nearly 100% of individuals will become infected and develop clinical illness. The incubation period of measles is about 10 to 12 days. Malnourished children are at higher risk of developing complications and mortality from this infection [1].

Measles remained as one of the vaccine-preventable diseases still causing major mortality and morbidity in developing countries. The severity of disease is higher among infants and adults than children and results complications from viral replication or bacterial superinfection including otitis media, pneumonia, laryngo-tracheo-bronchitis (croup), diarrhoea, encephalitis and blindness [2].

Global measles control has been very successful. Worldwide, an estimated 60% measles-associated mortality reduction with 75% reduction in the African region were achieved following implementation of the 2001 World Health Organization (WHO) member states goal of 50% measles mortality reduction by 2005, compared with the 1999 estimate [3–5]. Following this progress, the WHO African region approved a new goal in 2006 to achieve a 90% measles-associated mortality reduction by 2010, compared with the 2000 estimate. In 2008, reported measles cases had decreased by 93% and an estimated 92% measles-associated mortality reduction was found [4, 5].

The African region technical advisory group (TAG) reviewed the measles control progress and set a new measles reduction target called a pre-elimination goal, aimed to reduce the annual incidence to <5 measles cases per million population in all African countries [1, 6]. National and sub-national level measles data analysis was put as a vital way to make informed decisions on progress towards achieving this goal.

In 1980, Ethiopia introduced measles vaccination as part of the routine extended program of immunization (EPI) with first dose administration at 9 months of age. The measles vaccine coverage remained below 50% until 2003. Following augmented efforts to improve performance by the Federal Ministry of Health (FMOH) and EPI support partners, administrative measles vaccination coverage improved from 44% in 2003 to 81% in 2010 at the national level [7].

In 2014, there were 114,900 measles deaths globally [8]. In 2013, an estimated 21.5 million children were not vaccinated against measles at the 9^th^ month of age. Of these, more than 60% children were from India (6.4 million), Nigeria (2.7 million), Pakistan (1.7 million), Ethiopia (1.1 million), Indonesia (0.7 million) and Democratic Republic of Congo (0.7 million). More than 70% of the global measles-related deaths occurred in these 6 countries in 2013 showing developing countries affected utmost [9].

Although the vaccination coverage is increasing from year to year in Ethiopia, the country continued reporting a higher number of measles cases and outbreaks in all regional states and city administrations. According to WHO, Ethiopia is experiencing an ongoing measles outbreak by reporting more than 14,000 confirmed cases in 2014 only [10] and being one of the world countries that experienced large outbreaks in 2009 and 2010 according to CDC report [11]. The purpose of this study is to characterize the epidemiology measles virus with greater emphasis on laboratory-confirmed cases and to provide evidence for the decision-making process in an effort to eliminate measles in the Southern Nations Nationalities and Peoples’ Region (SNNPR) of Ethiopia.

## Methods

### Study setting

**Fig. 1:**
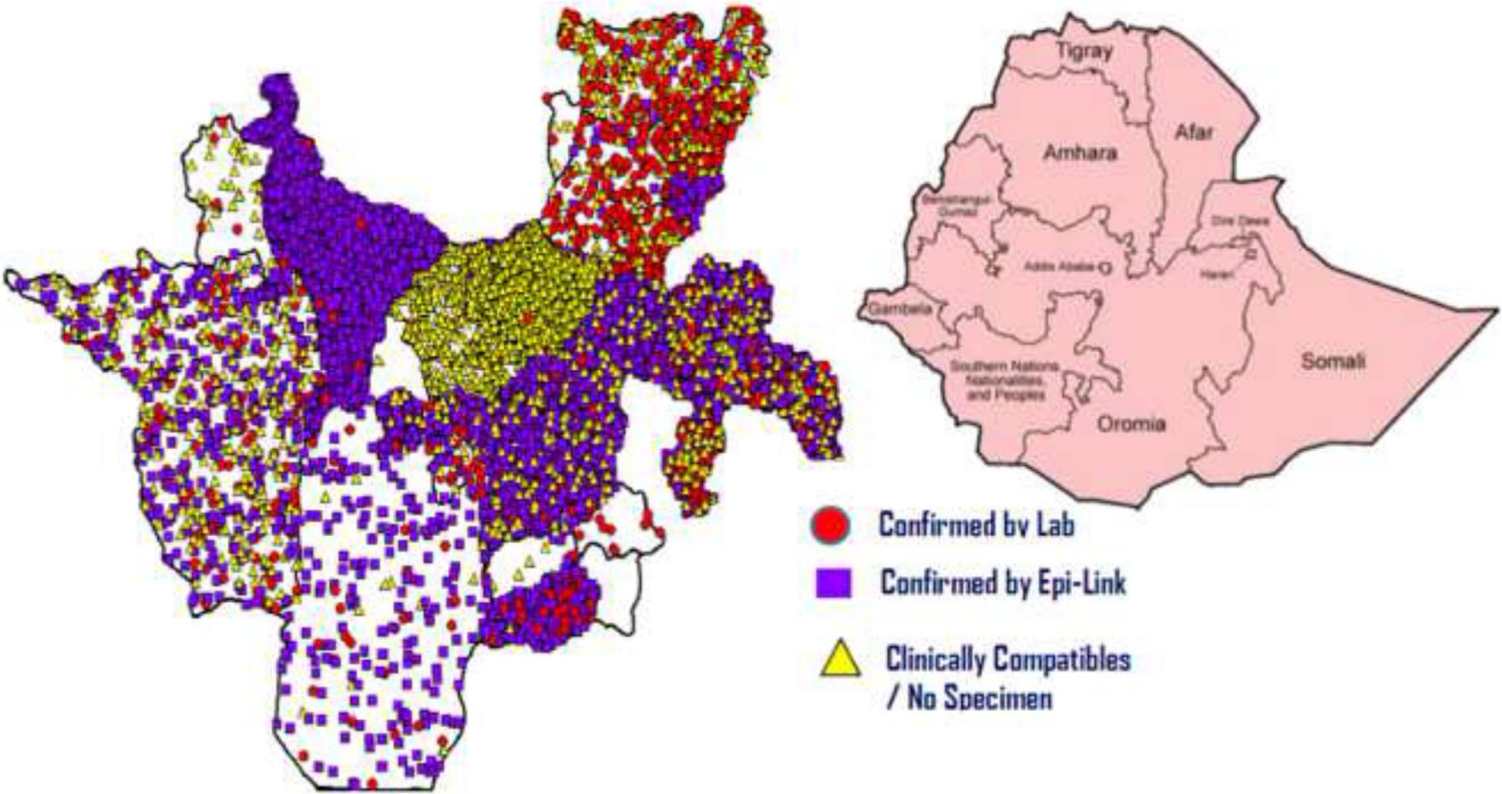
Map of Ethiopia Showing the Relative Location of SNNPR and its Administrative Zones with Distribution of Measles Cases (Source: WIKIPEDIA, the free Encyclopedia at https://en.wikipedia.org/wiki/Regions_of_Ethiopia)

The study was carried out in SNNPR. SNNPR is the third most populous region in Ethiopia, with a population of 15 million, a growth rate of 2.9 per annum and a child mortality rate of 85 per 1000 livebirths in 2007 and the region covers an estimated area of 105,887.18 square kilometers, [12]. The SNNPR is located in South Western part of Ethiopia. Administratively, the region is divided in to zones, that further divided as woredas and kebeles. The region shares common boundaries with Oromia in Eastern and Northern, Gambella in West, South Sudan Republic in South-Western and Kenya in Southern (Fig. 1).

### Study population and case definition

The WHO African Regional Office/AfRO measles case-based surveillance guidelines were adjusted for use in Ethiopia as well in SNNPR as of 2004. All age groups were included in this study using the case definition. A suspected measles case is defined as any person with generalized maculopapular rash and fever plus one of the following: cough or coryza (runny nose) or conjunctivitis (red eyes) or any person a clinician suspects to have measles. Laboratory confirmed measles case is a case that has recent measles virus-specific immunoglobulin M (IgM) antibody in blood by serological test and did not receive a measles vaccine within the last 30 days. Confirmed measles by epidemiological linkage is a measles case from which a specimen had not taken for serologic confirmation but linked (in place, person and time) to lab-confirmed cases. Clinically confirmed/compatible cases are individuals without blood and no epi-linkage with lab-confirmed cases or have equivocal measles IgM test results in blood, this will be high in seasons of no lab testing. Discarded cases are individuals with negative measles IgM lab result or IgM positive with a vaccination history within 2 weeks of blood drawn. Measles outbreaks are defined as the occurrence of 3 or more lab-confirmed measles cases reported from the same district or catchment area of a health facility with rash onset within 4 weeks. Incidence is the total number of confirmed measles cases by lab confirmation and epidemiological linkage per million population [1].

### Specimen collection and transportation

At first contact with suspected cases, about 5 ml blood was drawn by venipuncture into a sterile anticoagulant-free tube. Almost all (99.6%) samples in this study were collected within 28 days of rash onset from suspected cases. Serum was separated from whole blood and transferred aseptically to a sterile vial. The serum specimens with completed case reporting form (CRF) were transported in cold boxes to the testing laboratory, National Measles Laboratory is located in Ethiopian Public Health Institute (EPHI), Addis Ababa, Ethiopia.

### Laboratory method and quality assurance

Enzyme-linked immunosorbent assay (ELISA) technique was done for measles-specific IgM antibody identification according to the manufacturer’s protocol (Enzygnost® Anti-Measles-Virus/IgM kit; Siemens Diagnostics Products, Marburg, Germany). Samples and reagents were manually dispensed using a micropipette (Gilson S.A.S., France). A 5 micro liter (μl) serum was diluted with 205 μl working solution (diluted blue solution and reconstituted rheumatoid factor). 150 μl of this diluted solution was transferred to a double (antigen coated and control) well of ELISA plate and incubated for an hour to allow patient’s antibody (if any) bind antigen coated surface. Test plates were washed using a microplate washer (Denley Instruments Ltd, England) to remove unbound antibody. 100 μl of enzyme-labeled antihuman IgM working solution was added to each well and incubated for an hour to allow the attachment of enzyme-labeled antibody with patient’s antibody, then washed to remove unbound antihuman IgM. 100 μl chromogen substrate solution was added to each well and allowed 30 min for enzyme labeled antibody (if any) to break the substrate and produce a color change. The optical densities (OD) were read at 450 nm with a 630-nm reference filter using an ELISA reader (Labsystems, Finland). The change in Od was found by subtracting the OD of the control well from OD of antigen well. Samples with a change in OD <0.1 were recoded negative and >0.2 were positive and equivocal when OD between 0.1 and 0.2. Samples with equivocal results were retested to be reported. Based on the surveillance protocol, samples with equivocal or negative measles IgM result were further tested for rubella virus IgM.

The laboratory was evaluated regularly for quality control, sends 10% quality control (QC) samples quarterly, receives external quality assurance (EQA) proficiency test (PT) samples annually and accredited yearly by WHO Global Measles and Rubella Laboratory Network for the purpose of generating credible lab results for the program. Job aids and standard operational procedure (SOP) were available in lab.

During this study period, the lab maintain its accreditation, scored ≥ 95% accuracy for both QC and PT samples and applied internal and external control samples with each run. Patient results were reported when the run was valid based on the controls used. Due to kit shortage, samples collected after mid October 2014 were not tested for measles IgM antibody.

### Data entry and analysis

Patient information and laboratory results were entered into Epi-Info database. In the surveillance, data were shared every Fri-day to the FMOH, WHO-Ethiopia, WHO-AfRO and all concerned for action . Data for this study purpose, 2007–2014, were extracted and analyzed using Epi-Info (Version 3.5.4. CDC, Atlanta, USA).

## Results

A total of 4810 suspected patients were notified with blood and tested in the lab during 2007–2014 from SNNPR State of Ethiopia. The mean age of study participants was 6.3 years ranging from a month to 84 years with 51.6% males. Evidence on sex was unavailable for 20 cases. The highest proportion of tested persons (36%) were children 1–4 years age followed by children 5–9 years (29.3%). The highest number of samples (976) were collected in 2013 and the lowest (466) in 2007 (Table 1). The highest (656) and lowest (208) number of cases had onset in January and September respectively. A higher number of samples were collected from Sidama (912) and Guraghe (700) zones of SNNPR, Ethiopia.

**Table 1:** Distribution of Measles Tested Cases in Different Variables in SNNPR, 2007-2014

Variables	Measles IgM Test Result				Total
	Category	Positive	Negative	Equivocal	
Sex	Male	788	1638	55	2481
	Female	711	1546	52	2309
	Unavailable	8	12	0	20
Age Group	<1 Year	113	389	11	513
	1–4 Years	469	1228	33	1730
	5–9 Years	412	975	24	1411
	10–14 Years	300	391	25	716
	≥15 Years	184	164	10	358
	Missed	29	49	4	82
Years	2007	137	316	13	466
	2008	77	449	18	544
	2009	85	393	6	484
	2010	255	343	17	615
	2011	203	282	14	499
	2012	107	546	10	663
	2013	348	611	17	976
	2014	295	256	12	563
Total		1507	3196	107	4810

**Fig. 2:**
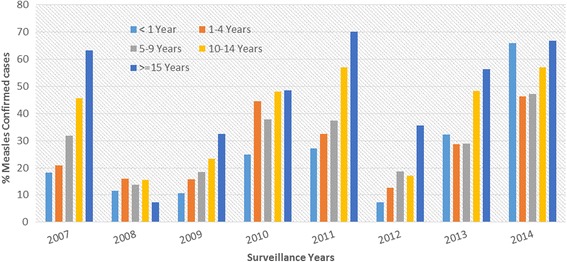
Proportion (%) of Lab Confirmed Measles by Age Group of Patients, SNNPR, Ethiopia, 2007–2014

Among tested samples, a total of 1507 (31.3%) samples were found positive for measles-specific IgM and the rest 3196 (66.4%) and 107 (2.2%) were negatives and equivocal (compatibles) respectively (Table 1). Of 1507 laboratory confirmed measles cases, 52.3% (788) were males and sex was unavailable for 8 (0.5%) cases. The lowest number of positives (77) and positive proportions (14.2%) were found in 2008 and the highest number of positives (348) were found in 2013. The highest proportion of positives (52.4%) was found in 2014 (Fig. 2).

**Fig. 3:**
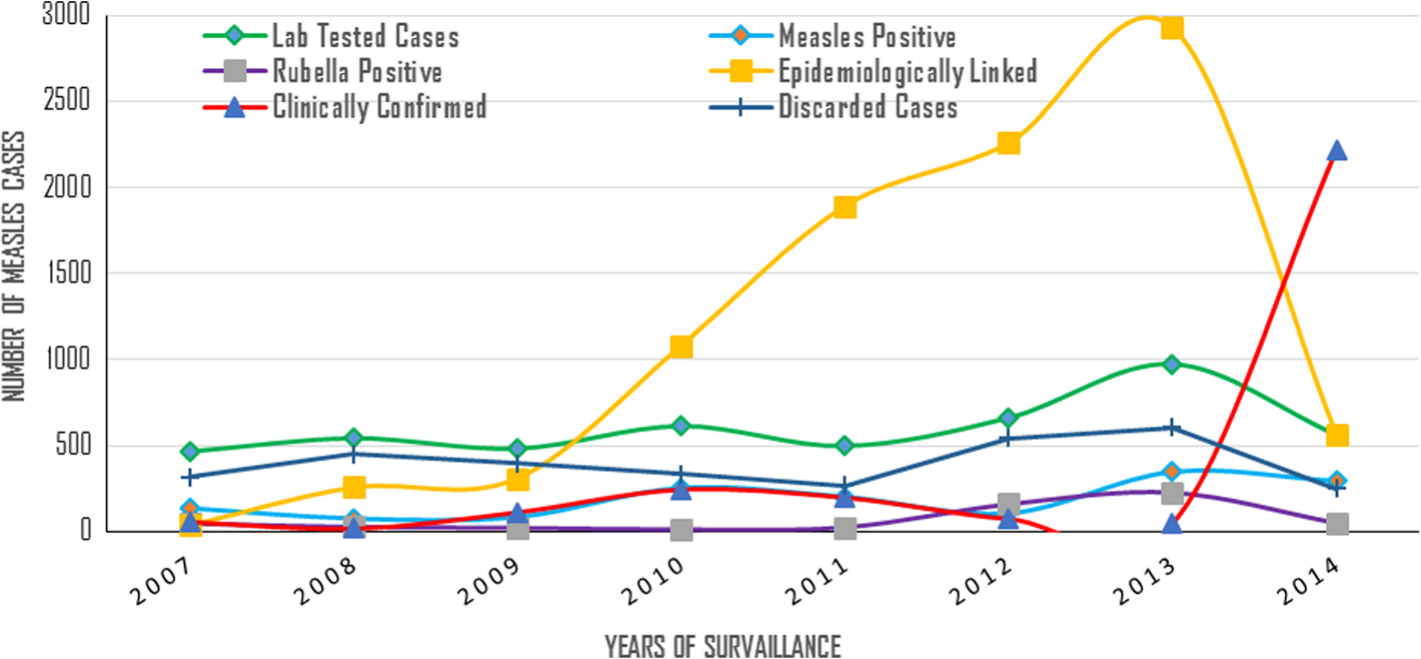
Distribution of Lab Tested Persons, Lab Confirmed Measles/Rubella, Epidemiologically Linked, Clinically Confirmed and Discarded Measles Cases, SNNPR, Ethiopia, 2007–2014

Among the total 3,303 measles IgM negatives and equivocal, 572 (17.3%) were found positive for rubella virus-specific IgM antibody, another rash febrile illness. Most of the rubella positive cases were found in the period 2012–2014 with no zero reports in all the study years (13 – 227 reports) (Fig. 3).

**Fig. 4:**
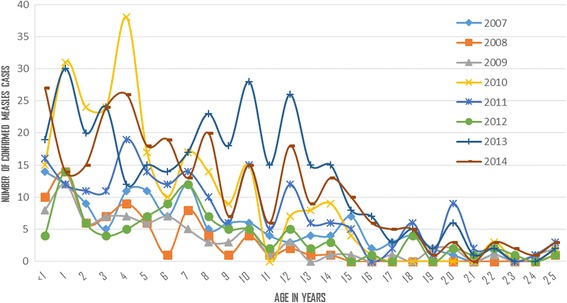
Distribution of Lab Confirmed Measles by Age of Patients in Years, SNNPR, Ethiopia, 2007–2014

Most of the measles cases occurred among children less than 15 years (Fig. 4). Likewise, rubella virus specific IgM antibody was detected among non-measles positive cases and was found higher from children up to 15 years of age (data not shown). Both measles and rubella confirmed cases decrease after the age of 15 years.

Although measles was most common among children less than 15 years of age, the highest and lowest positive proportions (%) were found from patients above 15 years and infants respectively throughout the study period (Fig. 2).

**Fig. 5:**
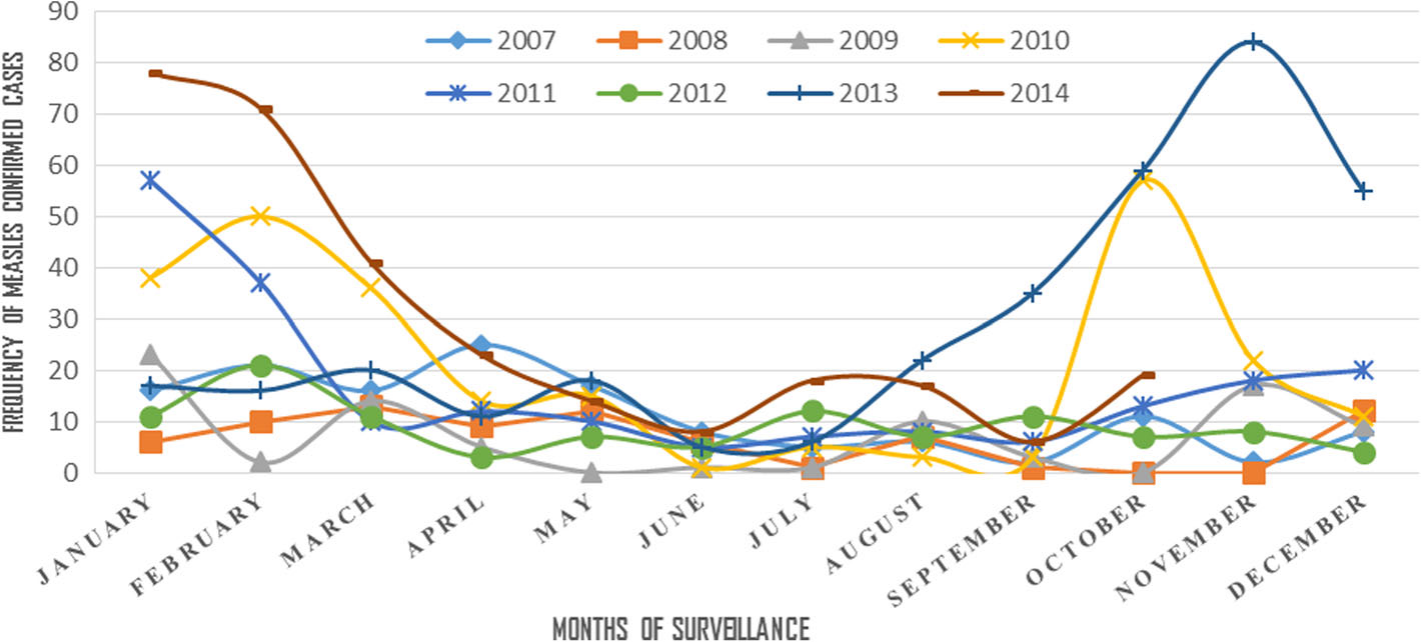
Distribution of Lab Confirmed Measles Patients by Study Years and Months, SNNPR, Ethiopia, 2007–2014

Measles was found endemic throughout the year and all zones of the region with the highest number (246) of positives in January and the lowest (39) in June during the study period (Fig. 5).

Similar to the laboratory confirmed cases, the epidemiologically linked measles cases increased linearly through the study period, from 36 cases in 2007 to 2928 in 2013 and the highest number of clinically confirmed cases were in 2014 (Fig. 3).

**Fig. 6:**
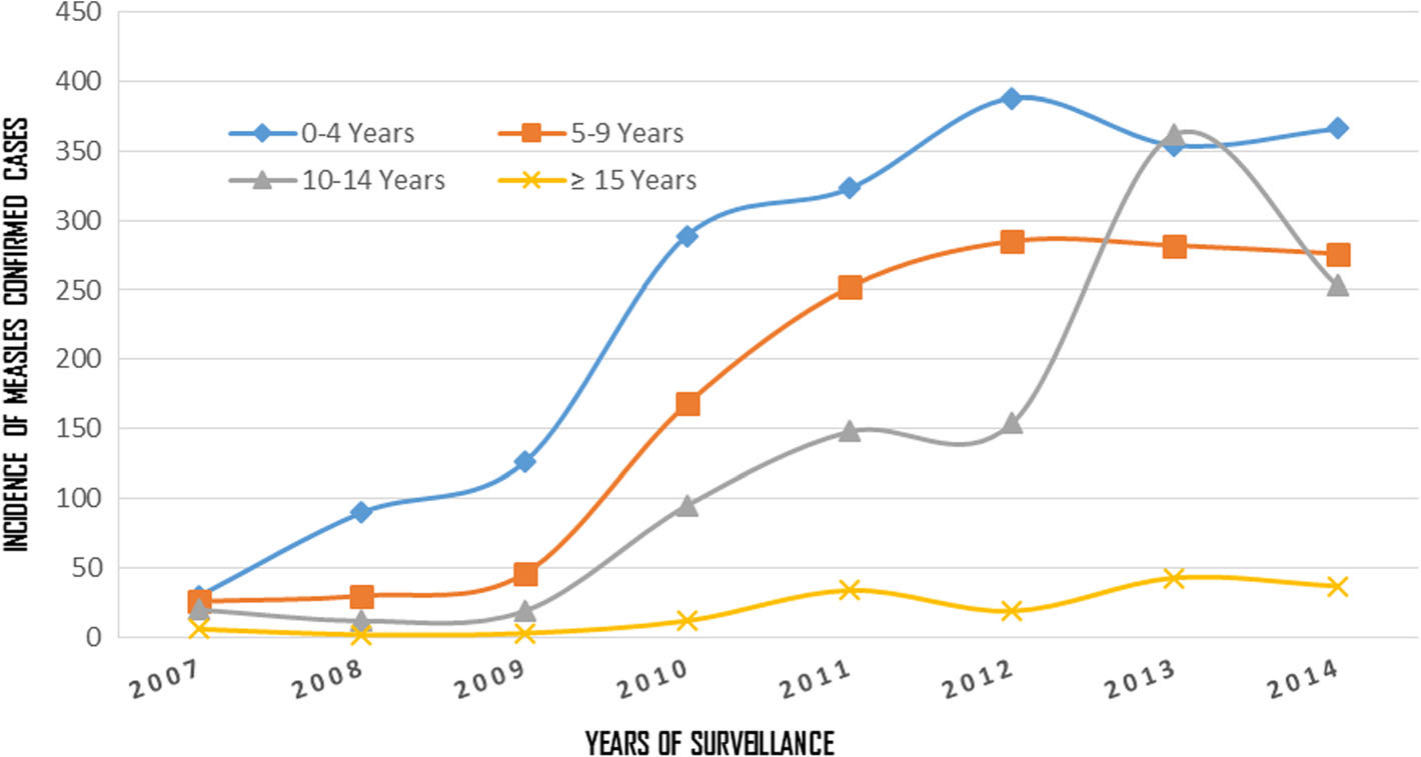
Incidence of Measles Confirmed Cases per million Population among Different Age Groups in SNNPR, Ethiopia, 2007–2014

In this study, the incidence of measles cases was found increasing among all age groups with a total of 15 in 2007 to 180 in 2013 per million population and the incidence decreased as the patient age increased in SNNPR (Fig. 6).

## Discussion

Analysis of the measles case-based surveillance laboratory data in SNNPR, Ethiopia for 2007–2014 revealed that 4810 suspected cases were investigated with an average 601 blood samples collected and tested per year. This was much lower than the mean number of annually notified cases of 1637 South West Nigeria, although the population is also double of SNNPR [13].

Of the laboratory tested cases, 31.3% were confirmed measles, showing the circulation of measles virus in the community or improved case capturing due to case sensitive surveillance in the region. This positivity rate was much higher than a study finding from South West Nigeria [13] and Zimbabwe [14]. The incidence of measles cases was found decreased as the age group increased, this is expected as measles infection will result in life-long immunity.

The number and proportion of lab-confirmed measles cases were found fluctuating but the incidence of confirmed measles (lab plus epi-linked) cases was found increasing strictly in the study years, 15 in 2007 to 180 in 2013 (higher than the pre-elimination target of <5 cases per million population, Fig. 6). Laboratory confirmed cases increased from 137 in 2007 to 295 in 2014, but lower in 2012. The frequency of measles cases fluctuated between different age groups (Fig. 4). This might be related to inconsistency in the Supplemental Immunization Activities (SIAs) coverage and time interval conducted and age of targeted children for vaccination. This was similar finding with a study in Nigeria where a pattern of high incidence followed by a low incidence was common [3].

Similar to the finding of a study from South West Nigeria [13], the highest number of measles confirmed cases occurred among children 1–4 years followed by 5–9 years children (Table 1, Fig. 6). Although the highest proportion (%) of measles positive cases was found among young adults (aged ≥ 15 years) each year (Fig. 2), the incidence of measles found among this age group was lowest (Fig. 6). Measles in the adults suggests an immunity gap likely due to the accumulation of susceptible individuals over several years from sub-optimal vaccination coverage in the previous years.

Most of the lab-confirmed measles cases occurred in the dry season (October-February), with the peak in January and February. This is in line with an earlier study finding from Abia State of Nigeria [15] and WHO report of measles infection trend [16]. This might be related with the more cultural gatherings (weddings, holydays) in this season that favors measles transmission in the SNNPR of Ethiopia.

According to WHO-AfRO Measles and Rubella surveillance guideline, measles suspected cases that were tested and had negative or equivocal result will be tested for rubella virus-specific IgM, another rash febrile illness. In this study, 17.3% of non-measles cases were found positive for rubella virus and children 5–9 years were affected greatly. This finding was much lower than a 37.6% positivity rate of rubella from Zimbabwe [17]. In general, 43.2% of laboratory tested samples were positive for either measles or rubella. The higher proportion of samples (56.8%) were found negative or equivocal for both measles and rubella. This may be due to other febrile rash illnesses in the community by infectious and non-infectious agents.

The highest number of clinically confirmed and the lowest number of epidemiologically linked measles cases in 2014 was due to a shortage of kit in the fourth quarter and the incidence of measles cases was seen dropped in this year as no sample was tested after the mid of October (Figs. 3 and 6). This obviously true that as the lab testing stops, clinical classification of cases will be increased and there will be no epidemiologically linked cases in this season.

In general, decreased incidence is normal to increasing vaccine coverage report of a specific disease. Unfortunately in SNNPR both reports of administrative measles vaccine coverage and measles incidence were found increasing at the same time [18]. This may be related to the improved case detection through time due to increased coverage, quality, and sensitivity as part of massive health service expansion in Ethiopia as well in SNNPR [19]. The efficacy and cold chain condition of vaccine needs further investigation in such high coverage reports and increasing case detection.

Our study had the following limitations. First, data presented here was mainly related to the lab-confirmed measles cases giving little emphasis to clinically confirmed and epi-linked cases as the details of these not submitted to the lab. Second, there was no data on the clinical presentation and severity of cases, vaccination status and case fatality rate as these information rarely submitted to the laboratory.

## Conclusion

In SNNPR, measles continued as an important public health problem. The incidence of confirmed measles was found increasing from year to year mostly affecting children aged a month to 4 years. Measles is a seasonal infection reaching a peak during January and February. We recommend wide age range measles campaign for the region and additional study to understand the knowledge, attitude, and practice of the professionals and the population for measles infection and vaccination is advisable. Finally genotyping of circulating measles virus strain is paramount important.

## Abbreviations

°C: Degree Celsius
CRF: Case reporting form
ELISA: Enzyme-linked immune-sorbent assay
EPHI: Ethiopian Public Health Institute
EPI: Extended Program of Immunization
EQA: External quality assessment
FMOH: Federal Ministry of Health
IgM: Immuno-globulin M
OD: Optical density
PT: Proficiency test
QC: Quality control
SERO: Scientific and Ethical Review Office
SNNPR: Southern Nation Nationalities and Peoples Region
SOP: Standard operating procedure
TAG: Technical Advisory Group
WHO-AfRO: World Health Organization African Regional Office
μl: micro-liter
